# Emission in the Biological Window from AIE-Based Carbazole-Substituted Furan-Based Compounds for Organic Light-Emitting Diodes and Random Lasers

**DOI:** 10.1021/acsomega.4c05484

**Published:** 2024-09-18

**Authors:** Kamila Lupinska, Sonia Kotowicz, Anna Grabarz, Mariola Siwy, Karolina Sulowska, Sebastian Mackowski, Lulu Bu, Yann Bretonnière, Chantal Andraud, Ewa Schab-Balcerzak, Lech Sznitko

**Affiliations:** † Institute of Advanced Materials, Faculty of Chemistry, Wroclaw University of Science and Technology, Wybrzeze Wyspianskiego 27, 50-370 Wroclaw, Poland; ‡ Institute of Chemistry, University of Silesia, 9 Szkolna Str., 40-006 Katowice, Poland; § Department of Physical and Theoretical Chemistry, Faculty of Natural Sciences, Comenius University, Ilkovičova 6, 84215 Bratislava, Slovakia; ∥ Centre of Polymer and Carbon Materials, Polish Academy of Sciences, 34 M. Curie-Sklodowska Str., 41-819 Zabrze, Poland; ⊥ Institute of Physics, Faculty of Physics, Astronomy and Informatics, Nicolaus Copernicus University, 5 Grudziadzka Str., 87-100 Torun, Poland; # Univ Lyon, Ens de Lyon, CNRS UMR 5182, Laboratoire de Chimie, F69342 Lyon, France

## Abstract

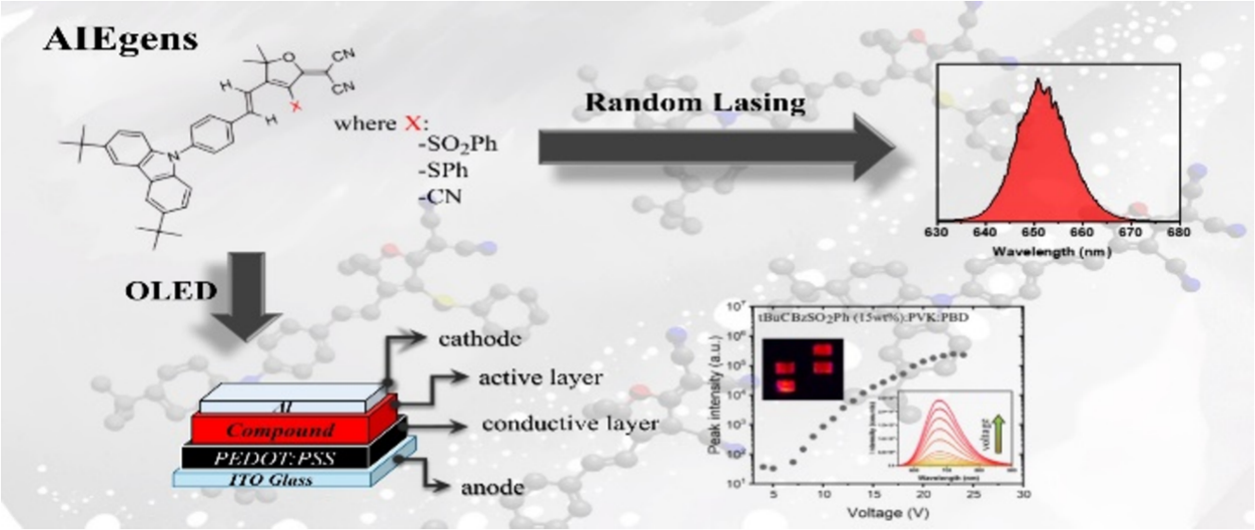

The emission quenching observed in devices utilizing luminescent materials such as solid thin films is a prevalent issue. Consequently, searching for new organic luminescent compounds exhibiting aggregation-induced emission (AIE) behavior and characterized by relatively simple and cost-effective synthesis is of crucial interest among applications from optoelectronics and organic lasing branches. Herein, we report the optical properties of three furan-based carbazole-substituted compounds, namely, tBuCBzSO_2_Ph, tBuCBzSPh, and tBuCbzTCF, exhibiting the aforementioned AIE phenomenon. The optical properties of dyes were determined in classical spectroscopic experiments supported by quantum-chemical calculations. The thermal investigations and electrochemical properties of dyes were performed to verify their usefulness in the construction of organic light-emitting diodes (OLEDs). In pursuit of this objective, OLEDs with a different design were fabricated, and their performance was subject to evaluation. In more detail, the different design strategies relying on the utilization of neat-dye films, as well as the preparation of dye-doped poly(9-vinylcarbazole):2-(4-*tert*-butylphenyl)-5-(4-biphenylyl)-1,3,4-oxadiazole (PVK:PBD) matrices were examined. The analysis that was conducted indicated the superior potential of tBuCBzSPh for optoelectronic applications. Notably, the positive impact of the AIE effect on the emission of the OLEDs and the ability to establish the lasing phenomenon in asymmetric, poly(methyl methacrylate) (PMMA)-doped polymeric slab waveguides were verified. The study showed that the combination of the strong intramolecular charge transfer (ICT) effect with dye aggregation enables the tuning of the emission of the OLED toward the first biological window, making examined dyes promising candidates for biomedical purposes. The same optical region can be attained for laser emission at relatively low pumping conditions, reaching as low as 7.3 kW of optical power for the tBuCBzSO_2_Ph compound.

## Introduction

In the past few years, small organic compounds emitting in the near-infrared (NIR) region of the radiation have gained attention from various fields of research. In this scope, considerable efforts have been made to explore new implementations of these compounds. Especially organic light-emitting diodes (OLEDs) and random lasers (RL) are particularly interesting to scientists because of their potential use in biological applications as light sources with excellent biocompatibility and flexibility.^1,2^ For instance, OLED technology can be adapted in biomedicine for lab-on-chip,^3,4^ optogenetic,^5,6^ therapeutic treatment,^7,8^ and health monitoring or biosensor applications.^9−11^ On the other hand, the implementation of RL in the biomedical field is also well-known and commonly used.^12−14^ Besides, an additional advantage arising from both of these kinds of light-emitting devices is the uncomplicated fabrication process and low production cost.^15^

Among the various techniques used to fabricate high-performance electronic devices, one relies on the utilization of organic compounds containing carbazole units in the structure.^16−19^ The aforementioned class of dyes demonstrates good thermal stability, high triplet energy, and efficient hole transport, which are all reported to have desirable properties.^20^ Consequently, molecules containing the carbazole group are widely utilized for fabricating OLEDs,^21,22^ organic field effect transistors (OFETs),^23,24^ and organic–inorganic hybrid perovskite solar cells (PSCs).^25,26^

OLED development mainly depends on the fabrication and functionalization of thin polymer films, which are used as the active matrix in the devices.^27−29^ Additionally, it should be emphasized that polymer thin films play a crucial role in achieving and enhancing laser emission.^30^ Unfortunately, many dyes showing excellent emission properties in a solvent medium experience severe emission quenching during aggregation (aggregation-induced quenching – ACQ).^31^ As a result of this detrimental effect, they can no longer be used as high-performance light-emitting organic devices. Consequently, luminescence quenching in aggregate states significantly restricts the progress of the OLED’s implementation. In this context, using aggregation-induced emission (AIE) compounds as active layers in organic optoelectronic materials represents an original and practical method to overcome these limitations.^32^ In the past, it has been demonstrated that the AIE phenomenon can have a positive impact on emission efficiency in OLED devices^33^ and low-efficiency roll-off.^34^ In particular, blue AIE-gens have been investigated and successfully applied in a two-color hybrid white OLED.^34^ The ACQ effect has also limited the growth of high-laser-gain materials and their development due to reduced luminescence efficiency and lower optical gain, resulting in difficulty in achieving efficient lasing. On the other hand, we recently have shown that AIE can positively impact laser light enhancement and can decrease the threshold of laser action.^35^

Despite the numerous advantages of OLEDs, it is worth adding that RL also garners great interest among different emitters. Nowadays, issues correlated with the external cavity occurring in various lasers lead to limitations of their application. RLs, on the other hand, rely on the scattering centers inside the sample, allowing optical feedback and thus obtaining laser action. From this point of view, compounds capable of random lasing (RLng) are promising materials in optical sensors,^36^ light therapy,^37^ display technologies,^38^ nanofiber-based photonic devices^39^ or in medicine, for cancer diagnostic applications.^40^

Different strategies to fabricate OLED devices and materials exhibiting RLng phenomena involve employing dyes containing two different groups with opposite functions in the molecules (i.e., electron-donating and withdrawing moieties), which can induce an intermolecular charge transfer (ICT) process.^41^ This effect can be promoted when both mentioned groups are connected by a bridge rich in π-electrons, enabling charge conductivity.^41^ In addition, the rapid deactivation of the excited molecule that occurs in ICT molecules has also been exploited in laser applications.^42^ Thus, small organic compounds with a donor-π–*a*cceptor (D-π–*A*) design are highly desirable in electrochemical and laser applications.^43−47^

**Figure 1:**
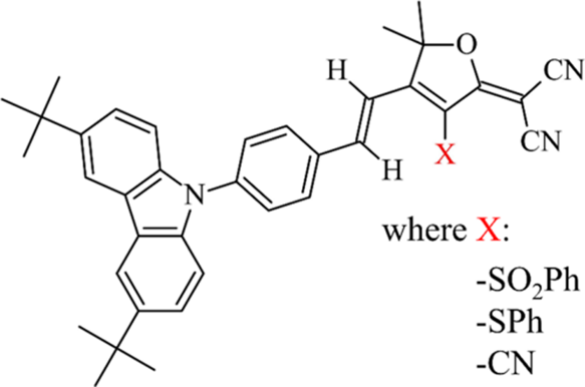
General formula of the studied dyes with hydrogen atoms is shown around the double carbon–carbon bond.

Herein, we focused on three compounds: tBuCBzSO_2_Ph (2-[4-[(1*E*)-2-[4-(3,6-Di*tert*-butyl-9*H*-carbazol-9-yl)phenyl]-ethenyl]-3-phenylsulfonyl-5,5-dimethyl-2(5*H*)-furanylidene]-propanedinitrile), tBuCBzSPh (2-[4-[(1*E*)-2-[4-(3,6-Di*tert*-butyl-9*H*-carbazol-9-yl)phenyl]-ethenyl]-3-phenylthio-5,5-dimethyl-2(5*H*)-furanylidene]-propanedinitrile), and tBuCBzTCF (2-[4-[(1*E*)-2-[4-(3,6-Di*tert*-butyl-9*H*-carbazol-9-yl)phenyl]-ethenyl]-3-cyano-5,5-dimethyl-2(5*H*)-furanylidene]-propanedinitrile) (see structures in Figure 1), containing carbazole units serving as a weak donor group and different furan derivatives responsible for withdrawing electrons (presenting a typical D-π–*A* molecular design). The compounds obtained for the need of this paper were originally synthesized, and their spectroscopic properties were described by Rémond et al.^48^ These compounds have been selected due to their emission spectral range appearing in the first biological window and their ICT nature, boosting their potential applications in optoelectronics and laser light generation. Each of these compounds shows optical features, such as emission, in the biological range or a high value of molar absorption coefficient, which is beneficial to achieving inversion on population and consequently to obtain lasing. The properties presented in the mentioned publication^48^ are desired in OLED applications and RLng-based light sources, while near-infrared (NIR) emission could be a key feature for further utilization in the developing biomedical industry. In addition, the synthesized dyes exhibit solid-state emission, suggesting that these molecules present active AIE properties.

## Experimental Section

### Sample Preparation

Liquid samples were prepared by dissolving selected compounds in a tetrahydrofuran (THF) solution, with the final concentration reaching approximately *C* ∼ 1.5 · 10^–5^ for the basic spectroscopic properties and *C* ∼ 1.5 · 10^–4^ for the AIE measurements. The synthesis of the studied compounds was reported under the procedure described in ref (48).

Thin polymeric films with poly(methyl methacrylate) (PMMA) were fabricated by dissolving 100 μL of dye of 1.15 mg/mL dye/THF solution into a 300 μL 2% PMMA/THF mixture to achieve a final concentration concerning the polymer mass of ∼2% compound/PMMA (absolute concentration for all samples were 1.92%). The PMMA was obtained from Sigma-Aldrich, and its molecular weight is 120 kDa. It was used as received. To obtain polymeric samples, we utilized the drop-casting technique and subsequently dried them in a THF-rich atmosphere to slow the evaporation process and enhance the quality of the resulting layers. The thickness values of the samples are 1.2 μm for SO2Ph, 3.7 μm for SPh, and 0.9 μm for –TCF.

These measurements were obtained using a deKTaK3 profilometer by averaging five measurements for each sample.

### Optical Characterization

The absorption and emission measurements were recorded using a Jasco V-670 UV–vis–NIR spectrometer and Horiba FluoroMax 4 fluorescence spectrophotometer, respectively. The AIE studies were performed using the same setup.

### Computational Details

All the DFT and time-dependent density functional theory (TD-DFT) calculations were performed with the latest version of Gaussian16 software.^49^ The standard optimization procedure was improved, i.e., the default self-consistent field convergence criterion was increased to 10^–10^ a.u., and the optimization threshold was enhanced to 10^–5^ a.u. on average residual forces. In all DFT and TD-DFT calculations, the so-called ultrafine pruned integration grid (99 radial shells and 590 angular points per shell) was adopted. The ground-state and excited-state geometries (optimized structure and related vibrational analysis) were established using the Pople 6-31G(d) basis set, while vertical transitions were characterized by adapting more extensive basis -6-311+G(2d,p). In the first step, ground-state geometric parameters were optimized and followed by vibrational analysis, which confirmed that found structures, in fact, correspond to true minima on ground-state potential energy surfaces (PES); later, analogous characterization was performed for excited-state structures. Following the results of a recent excited-state properties benchmark treating a large number of real-life dyes, MN15^50^ functional was chosen since it provides superior accuracy with respect to other well-known methods dedicated to charge transfer (CT) dyes (i.e., M06-2X, CAM-B3LYP or ωB97X-D).^51^

In order to account for the conditions of experimental measurements (here tetrahydrofuran solvent), all described calculations were performed using the polarizable continuum model (PCM)^52,53^ in its linear-response (LR)^54,55^ (optimization and vibrational analysis) or corrected-linear-response (cLR)^56^ (vertical electronic energies) variants.

Finally, electron density difference (EDD) plots, together with charge transfer parameters, were estimated to gain additional insight into the character of the analyzed transitions. The above results were obtained using PCM-TD-MN15/6-311+G(2d,p), a theory-level adopting procedure proposed by Le Bahers and co-workers.^100^ EDD plots were prepared using 0.002 a.u. In these graphs, the blue (red) areas indicate a density depletion (gain) upon photon absorption.

### Thermal Investigation

Differential scanning calorimetry (DSC) was performed using a Du Pont 1090B apparatus with a heating/cooling rate of 20 °C·min^–1^ under nitrogen and using aluminum sample pans in the range of 0–320 °C. The glass transition temperature (*T*_g_) was recorded in the second scan after cooling.

### Electrochemical Investigations

Electrochemical properties were investigated by cyclic voltammetry (CV) and differential pulse voltammetry (DPV). The results were registered on Eco Chemie AutolabPGSTAT128n potentiostat using the platinum electrode as the working electrode with 0.1 mol/dm^3^ Bu4NPF6 (Sigma-Aldrich) electrolyte and dichloromethane solution (Sigma-Aldrich) with 10^–3^ mol/dm^3^ concentration. The platinum coil and silver wire were used as the auxiliary and reference electrodes, respectively. The moderate scan rate for cyclic voltammetry and differential pulse voltammetry was equal to 100 mV/s. The solutions were purged with argon before every measurement and performed at 24 ± 1 °C. The ferrocene couple (Fc/Fc^+^) was used as the internal standard, and the EHOMO of Fc/Fc^+^ was calculated to be equal to −5.1 eV, as shown in the publication.^57^

### Organic Light-Emitting Diode

Devices with sandwich configurations ITO/PEDOT:PSS/compound/Al and ITO/PEDOT:PSS/compound:PVK:PBD/Al with 1, 2, and 15 wt % compound content in the blend were prepared. Devices were prepared on OSSILA substrates with pixilated ITO anodes and cleaned with detergent, deionized water, 10% NaOH solution, water, and isopropyl alcohol in an ultrasonic bath. Substrates were covered with PEDOT:PSS film by spin coating at 5000 rpm for 60 s and annealed for 5 min at 120 °C. The active layer was spin-coated on top of the PEDOT:PSS layer from a chloroform solution (10 mg/mL) at 1000 rpm for 60 s and annealed for 5 min at 100 °C. Finally, Al was vacuum-deposited at a pressure of 5 · 10^–5^ Torr. Electroluminescence (EL) spectra were measured with the voltage applied by using a precise voltage supply (GwInstek PSP-405), and the sample was fixed to an XYZ stage. Light from the OLED device was collected through a 30 mm lens, focused on the entrance slit (50 μm) of a monochromator (Shamrock SR-303i), and detected using a CCD detector (AndoriDus 12305). Typical acquisition times were equal to 10 s. The prealignment of the setup was done using a 405 nm laser.

### Random Lasing Measurements

For laser studies, we utilized the Surleite II Continuum third harmonic (λ = 355 nm) of a Nd:YAG nanosecond pulses laser (pulse duration τ = 5 ns, repetition rate *f* = 10 Hz) connected with the Optical Parametric Oscillator (Horizon, midband OPO by Continuum). The OPO was used to select the excitation wavelength corresponding to the maximum absorption for compounds tBuCBzSO2Ph, tBuCBzSPh, and tBuCBzTCF. The laser beam from the OPO was directed through to the half-wave plate and polarizer, which are used to keep the vertical and linear polarization of the beam and additionally to provide control over its intensity by rotation of the half-wave plate azimuth. Next, the excitation beam was carried out by a beam expanding system composed of two focusing lenses to form an “excitation stripe” and afterward fell on a regulated slit, responsible for the length of an excitation area. The samples were positioned behind the cylindrical lens focal point, which was placed close to the regulated slit. The laser emission from the probes was collected by a fiber connected to an Andor Shamrock SR-500i spectrometer equipped with an AndoriDus CCD camera. All of the measurements concerning lasing threshold determination were carried out for “excitation stripes” of 0.83 cm in length and 0.03 cm in height for all of the studied compounds.

## Results

This section is divided into parts. The first one describes the results of basic spectroscopic, thermal, and electrochemical analysis. The second section refers to potential applications for the development of OLEDs and laser device development.

### Basic Spectroscopic Properties and Thermal and Electrochemical Analysis

#### Quantum-Chemical Calculations

First, we aimed to explore quantum-chemical calculations to gain insight into the photophysics of the studied molecules and to verify the occurrence of ICT. The calculations were performed using the time-dependent density functional theory (TD-DFT) approach. All details about the computational protocol employed here are described in the Experimental Section.

Characterization of the investigated compounds started by checking whether they had more than one stable rotamer in the tetrahydrofuran (THF) solution by rotating the acceptor moiety (SO_2_Ph, SPh, and TCF) two stable structures for each compound were identified. In more detail, the most stable conformers have the functional groups (−SO_2_Ph, −SPh, and −CN, respectively) pointing “up” (for details, see the electron density difference (EED) plots in Table 2). The difference in Free Gibbs energy (Δ*G*) between respective rotamers ranges from 0.01 for the TCF dye up to 2.7 kcal/mol reported for the SO_2_Ph derivative (See Table S1 in SI). On the ground of Boltzmann distribution and assuming room temperature, both rotamers of tBuCBzTCF and tBuCBzSPh dyes can be present in the solution. In contrast, the probability of observing another conformer of tBuCBzSO_2_Ph is negligible (≪1%). Consequently, two rotamers were included in the further analysis for tBuCBzTCF and tBuCBzSPh only.

**Figure 2:**
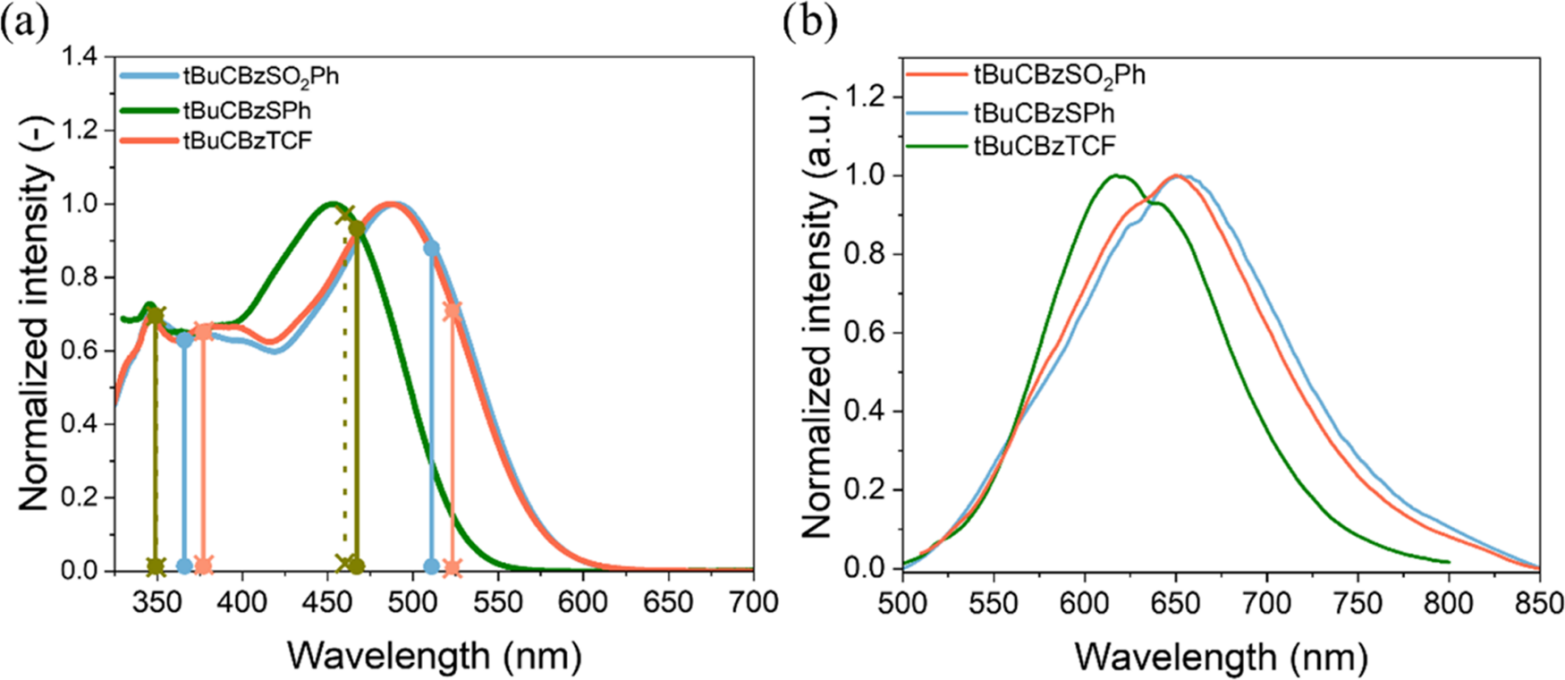
Experimental absorption (a) and emission (b) spectra of investigated compounds in THF solution *C* ∼ 1.5 × 10^–5^ mol/dm^3^. The emission spectra were recorded at room temperature using an excitation wavelength equal to the maximum absorption for each compound. Vertical lines indicate maxima computed for S0 → S1 and S0 → S3 transitions calculated by the TD-DFT approach (the details can be found in the Experimental Section). The continuous lines with dots at the ends represent more stable conformers, while dashed lines with crosses at the ends are less stable conformers. Inset in part a shows the molecular structure of investigated compounds.

The selected computational protocol correctly describes the photophysics of the studied compounds. TD-DFT results were compared with measured spectroscopic parameters (Table 1 and Figure 2). As depicted in Table 1, the MN15 functional can satisfactorily reproduce spectral features of absorption bands, including general shape as well as maxima position. Notably, the predicted position of absorption maxima is slightly overestimated (in the case of tBuCBzTCF to the larger extent, ca. 30 nm), yet the absolute errors (AE) are in the acceptable range of 0.04–0.11 eV. In turn, larger deviations from experimental values were observed for emission spectra, i.e., tBuCBzTCF and tBuCBzSO_2_Ph bands are ca. 50 nm (AE < 0.2 eV) underestimated while predicted tBuCBzSPh band position differs only negligibly (6 nm). Such discrepancies fully coincide with the strong charge transfer (CT) character of examined transitions. This overestimation in absorption and emission spectra may be caused by several reasons; for instance, the computational model assumes ideal conditions, whereas the complexity of the real systems might be higher. For example, the interactions with solvent were modeled using the PCM method, which is accurate only for averaged electrostatic interactions. Besides, compounds with charge transfer characteristics are also very sensitive to environmental changes like temperature or the solvent’s hygroscopic properties, etc., which may impact the measured absorption spectra. It is worth highlighting that we can observe the difference between two stable conformers in the THF for the SPh dye. Therefore, even the conformational differences might contribute to discrepancies in both values. Although some differences between the predicted and actual values are observed, the overall fit of the results is satisfactory.

**Table 1:** Experimental and Calculated Spectroscopic Parameters^a^,^b^

	theory							experiment				
compound	λ_abs_ (nm)	*f*	λ_abs_ (nm)	*f*	λ_emi_ (nm)	*f*	Δλ (nm)	λ_abs_ (nm)	ε (M^–1^ cm^–1^)	λ_emi_ (nm)	Δλ (nm)	fwhm (nm)
	S_0_ → S_1_	S_0_ → S_1_	S_0_ → S_3_	S_0_ → S_3_	S_1_ → S_0_	S_1_ → S_0_						
tBuCBzSO_2_Ph	**512**	**1.19**	**366**	**0.67**	**603**	**1.60**	**92**	489	51,500	653	164	126
tBuCBzSPh	**467**	**1.14**	**349**	**<0.05**	**612**	**1.52**	**145**	453	39,230	618	165	114
	460	1.11	349	0.39	554	1.53	94					
tBuCBzTCF	**523**	**1.15**	**377**	**0.50**	**595**	**1.62**	**72**	487	50,550	649	162	136
	523	1.15	377	0.50	598	1.54	75					

a In the theoretical part, the λ^abs^ and λ^emi^ are vertical energies computed at the MN15-cLR-PCM theory level. Δλ denotes the Stokes shifts.

b The values corresponding to more stable conformers are marked with bold font.

The TD-DFT analysis confirmed that the main absorption bands correspond to the S_0_ → S_1_ transitions, while weaker bands around 350 nm coincide with S_0_ → S_3_. Lowest-lying π–π* excitations of all of the examined structures are characterized by large oscillator strengths (varying from 1.11 to 1.19), while oscillator strengths corresponding to S_0_ → S_3_ transitions are significantly lower (0.39–0.67). For all dyes, the largest contributions in the main transitions can be ascribed to one-electron HOMO–LUMO excitation; however, non-negligible fractions from the HOMO–2 to LUMO transition are also present. As expected, the computational protocol attributed the emission bands to S_1_ → S_0_, but the ascribed oscillator strengths are much higher (1.52–1.62).

To gain insight into the nature of the lowest-lying π → π* transitions, we assessed related CT parameters. As can be seen from EED plots (see Table 2), the carbazole unit acts as an electron donor, while furan-based moieties serve as an electron acceptor for all examined derivatives, which is consistent with our preliminary assumptions concerning D-π–*A* design. Notably, no change in electron density is observed within tBu groups or phenyl rings attached to sulfur atoms. It should be highlighted that all examined transitions displayed a strong CT nature, which is reflected by the substantial change in the dipole moment (μ_ES_*-*μ_GS_), reaching above 20 D for tBuCBzSO_2_Ph and tBuCBzTCF as well as high total transferred charge (*q*_CT_) values. As can be seen, the donor and acceptor moieties of examined dyes are connected with an extended π-conjugated spacer; thus, all molecules are also characterized by high charge transfer distance (*d*_CT_) (>4 Å). Notably, among the series tBuCBzSPh presents significantly lower (yet still meaningful) CT parameters (μ_ES_*-*μ_GS_, *q*_CT_, and *d*_CT_) than the other two dyes.

**Table 2:**
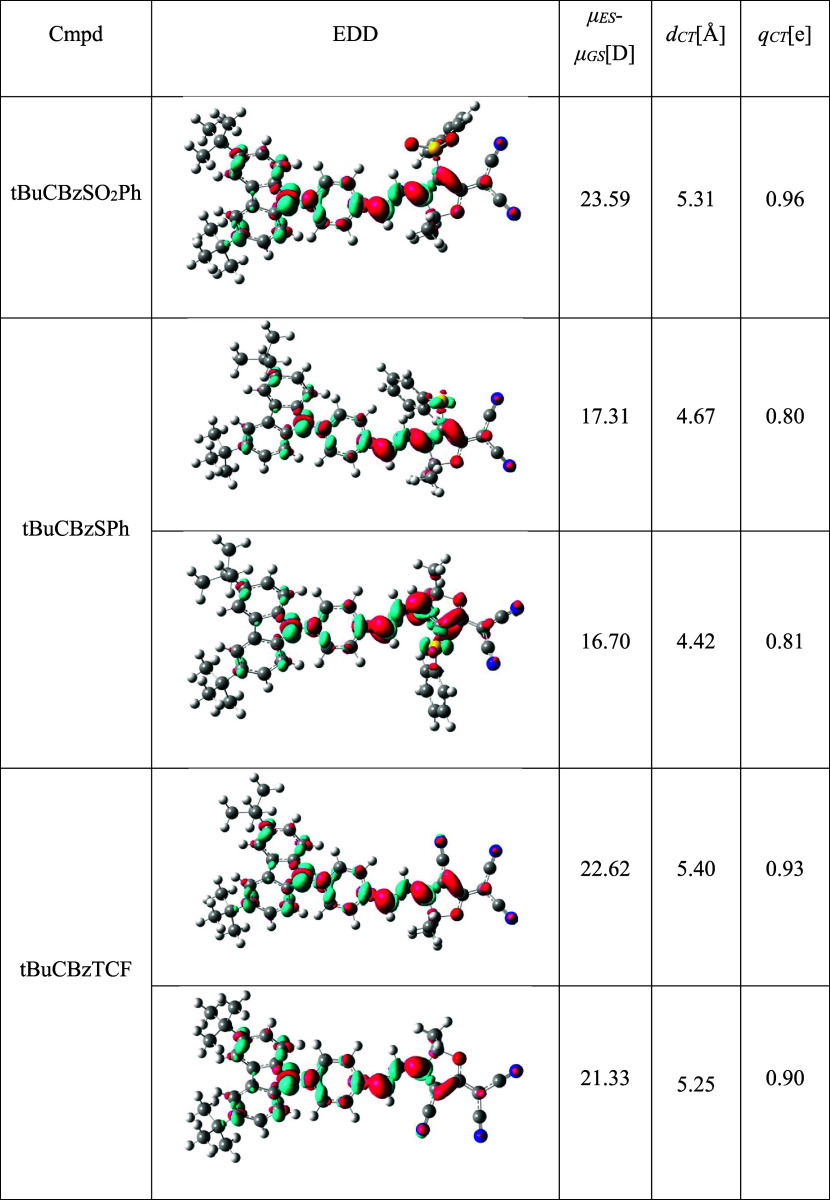
Electron Density Difference (EDD) Plots and Related S_0_ → S_1_ Electron Transition Parameters of Examined Compounds – *d*_CT_ Stands for Charge Transfer Distance, *q*_CT_ is the Total Transferred Charge, μ_ES_-μ_GS_ Represents the Difference in Dipole Moment Magnitude between the Ground and Franck–Condon Region of the Excited State^a^

a In the EDD, the blue (red) color marks electron density depletion (gain) upon photon absorption. The contour value was set to 0.002 a.u.

#### AIE

**Figure 3:**
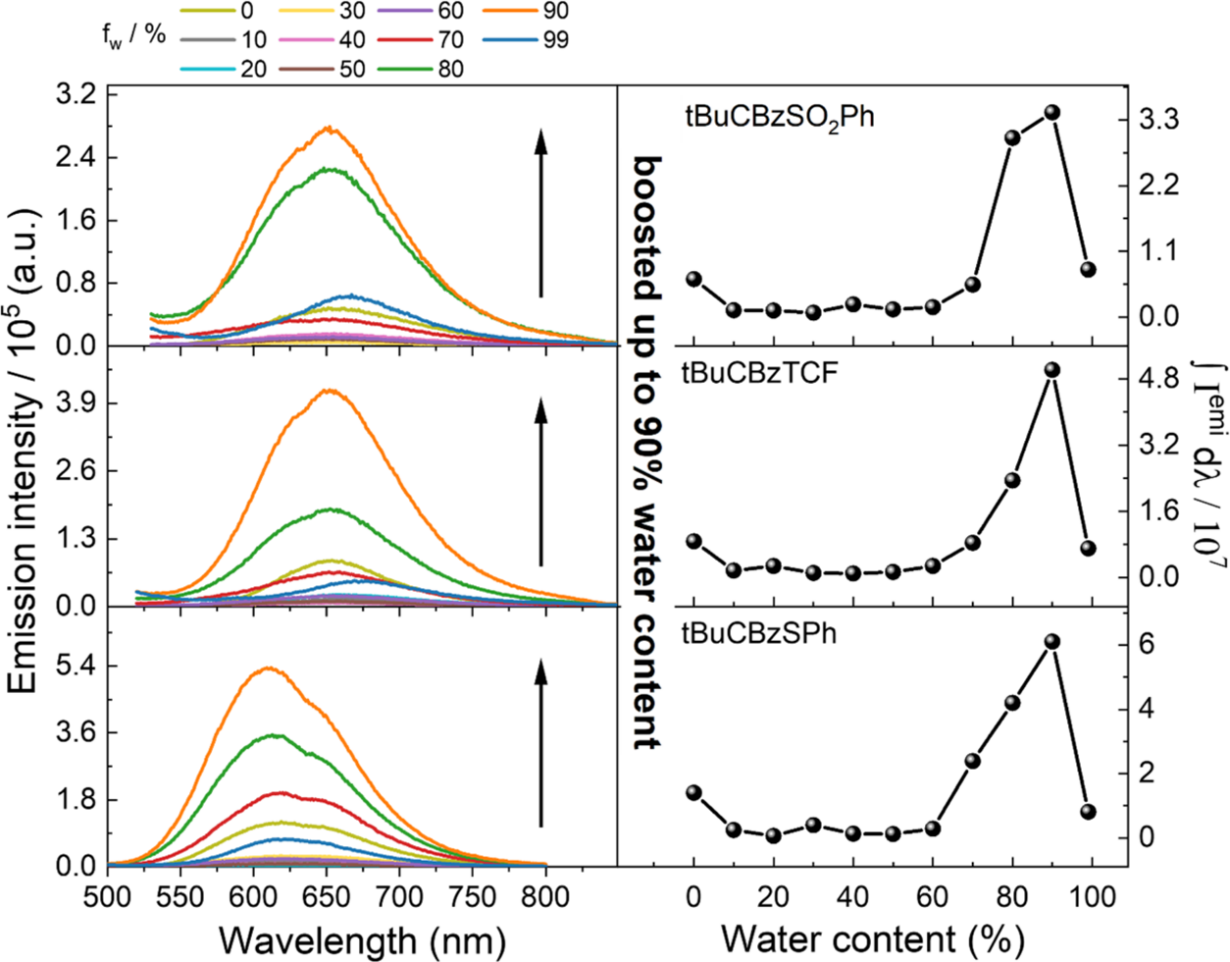
Impact of water addition on the emission intensity (left panel) and dependence on water. Water fraction (*f*_w_/%) on integrated emission spectra (∫I^emi^dλ) taken from the above (right panel) for the studied compounds.

To test whether the investigated compounds exhibit AIE, different fractions of THF solution (*C* ∼ 1.5 × 10^–4^ mol/dm^3^) were mixed with water. By increasing the water/THF ratio, aggregation of the investigated dyes was forced, as investigated dyes are not soluble in water.^58^Figure 3 presents the emission intensity and the evolution of photoluminescence spectra with respect to the water fractions (*f*_w_/%) for each compound. It is clearly visible that all compounds are AIE-active. The emission was recorded for excitation at the maximum of the absorption wavelength (see Table 1); the spectral emission was collected in the 550–850 nm range for the tBuCBzSO_2_Ph and tBuCBzSPh while for compound tBuCBzTCF from 550 to 800 nm. At the origin of the nonsolvent addition, we observed a slightly lower integrated intensity of the emission spectra (see Figure 3). It can be explained by the higher polarity of mixed solvents and dilution of the resulting dye/solvent/nonsolvent mixture.^59^ A significant enhancement in the emission intensity emerged at water content approaching 60–70%, depending on the investigated dye, appointing the threshold value for which aggregation occurs. Maximum emission intensities are reached when the water fraction is 90% for all three dyes, with enhancement factors equal to 5.4, 4.3, and 5.7 (relative to pure THF solution) for tBuCBzSO_2_Ph, tBuCBzSPh, and tBuCBzTCF, respectively. The emissions intensity decreases drastically for the 99% water/THF mixture due to a substantial quantity of nonsolvent and sedimentation processes.

Interestingly, for tBuCBzSO_2_Ph and tBuCBzTCF, we can see that emission in the AIE experiment is positioned around ∼650 nm. For the highest water addition (∼99%), we see a slight red emission shift in both cases with respect to no water content (Figure S1 in the SI). In the case of the tBuCBzSPh dye, the AIE behavior is also present; however, the explanation of the emission nature is not clear-cut. It is undoubtedly evident that in the last case aggregates play an important role in emissions intensity; nevertheless, the shape of the spectrum remains almost unchanged (see Figure S1 in SI).

#### Thermal Investigations

The thermal characteristics were determined using differential scanning calorimetry (DSC) as described in the Experimental Section. In the first heating scan, the endothermal peaks of the melting temperature (*T*_m_) in the 306–327 °C range were registered. The compound tBuCBSO_2_Ph was characterized by the highest *T*_m_. In the second heating scan, the glass transition temperature (*T*_g_) at 158 °C (tBuCBSO_2_Ph), 138 °C (tBuCBzSPh), and 223 °C (tBuCBzTCF) was detected. In the case of tBuCBzTCF, after *T*_g_, during further heating, the cold crystallization temperature (*T*_cc_ = 240 °C) and the melting temperature (*T*_m_ = 312 °C) were observed. For compounds tBuCBSO_2_Ph and tBuCBzSPh, the lack of *T*_cc_ and *T*_m_ was noticed. The presence of the glass transition temperature is characteristic of molecular glasses, where the amorphous glass is formed via a supercooled liquid of the crystalline compound (*T*_m_ in the first heating scan). Results from DSC measurements are shown in Figure S2 in the SI. To summarize, all compounds showed high melting and glass transition temperatures required for optoelectronic applications.

#### Electrochemical Properties

**Figure 4:**
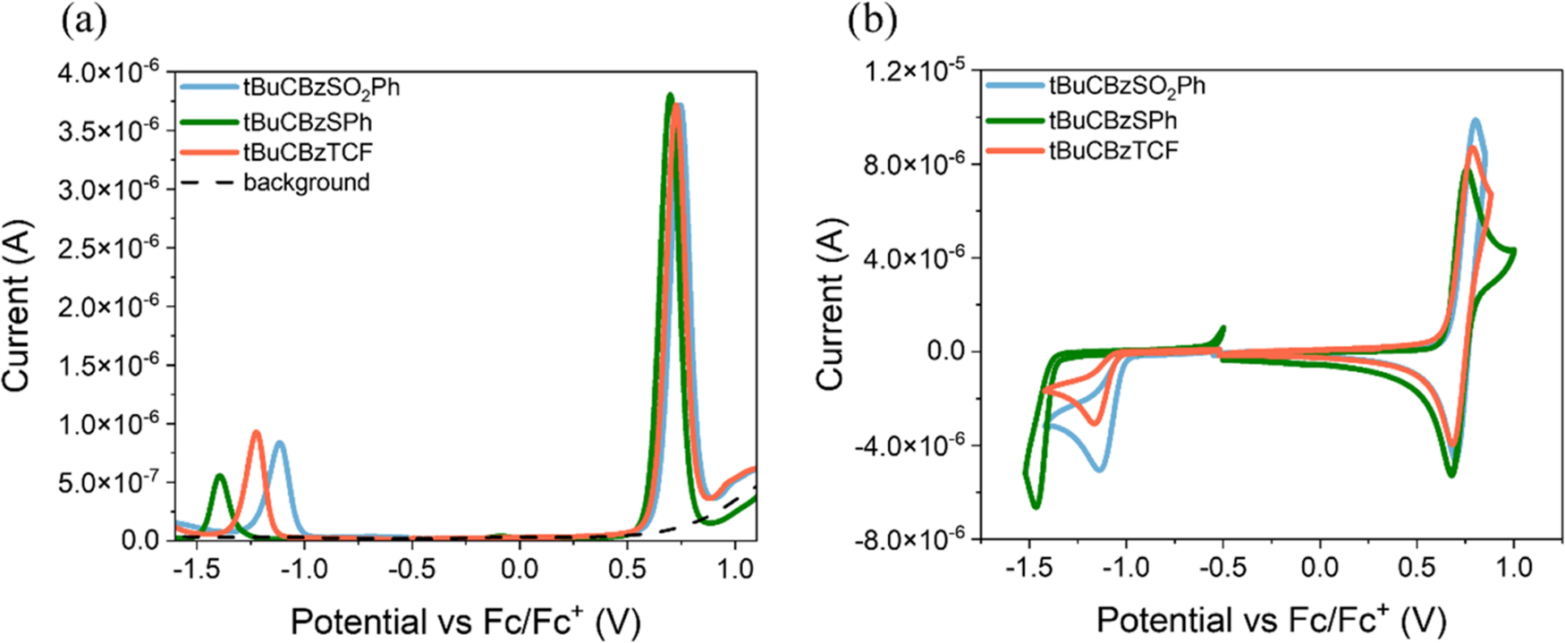
Voltammograms measured with DPV (a) and CV methods (b).

The electrochemical investigations were performed (as described in the Experimental Section) in a dichloromethane solution with a concentration equal to 10^–3^ and 10^–1^ mol/dm^3^ Bu_4_NPF_6_ electrolyte. The electrochemical data from the cyclic voltammetry (CV) and differential pulse voltammetry (DPV) measurements are collected in Table 3, and the voltammograms are presented in Figures 4 and S3. The onset potentials from the first oxidation and reduction processes were used to calculate the ionization potentials (IP) and electron affinities (EA).

**Table 3:** Electrochemical Data with the *E*_red_, *E*_ox_, and Onset Potentials (vs. Fc/Fc^+^) and an Energy Band Gap *E*_g_^a^

molecule	method	*E*_red_ [V]	*E*_red_^(onset)^ [V]	*E*_ox_ [V]	*E*_ox_^(onset)^ [V]	EA [eV]	IP [eV]	*E*_g_ [eV]
tBuCBzSO_2_Ph	CV	–1.13^b^	–1.01	0.80^c^	0.67	–4.09	–5.77	1.68
	DPV	–1.11	–1.00	0.74	0.65	–4.10	–5.75	1.65
tBuCBzSPh	CV	–1.46^b^	–1.38	0.76^c^	0.64	–3.72	–5.74	2.02
	DPV	–1.39	–1.31	0.69	0.61	–3.79	–5.71	1.92
tBuCBzTCF	CV	–1.16^b^	–1.05	0.78^c^	0.62	–4.05	–5.72	1.67
	DPV	–1.22	–1.13	0.73	0.57	–3.97	–5.67	1.70

a IP = (−5.1-*E*_ox_^(onset)^) · e^–^, EA = (−5.1-*E*_red_^(onset)^) · e^–^, *E*_g_ = *E*_ox_^(onset)^-*E*_red_^(onset)^. Solvent: CH_2_Cl_2_ with *c* = 10^–3^ mol/dm^3^ and electrolyte 0.1 mol/dm^3^ Bu_4_NPF_6_ and platinum wire (Pt) as a working electrode.

b Irreversible process.

c *Quasi*-reversible process. *E*_ox_– the first oxidation process, *E*_red_– the first reduction process, *E*_red_^(onset)^ – the onset potential of the first reduction process, *E*_ox_^(onset)^– the onset potential of the first oxidation process. IP and EA as ionization potential and electron affinity. The Fc/Fc^+^ was used as the internal standard.

The investigated compounds in the solution were electrochemically active, and the reduction and oxidation processes were registered. The first irreversible reduction process was noticed in the range of −1.46 to −1.11 V vs Fc/Fc^+^ (cf. Table 2), and the first quasi-reversible oxidation process was detected in the range of 0.80–0.69 V vs Fc/Fc^+^ for the studied dyes. The presence of the acceptor groups, such as −CH=C(CN)_2_ moieties, allowed the registration of the reduction process (cf. Figure 4). The reduction process was the easiest for tBuCBzSO_2_Ph and the most difficult for tBuCBzSPh. In the case of tBuCBzTCF, two reduction peaks were recorded, one at *E*_red_ = −1.22 V vs Fc/Fc^+^ and the other at *E*_red_ = −1.66 V vs Fc/Fc^+^(cf. Figure S3), which is associated with the presence of the third group −CN. The oxidation process is related to the presence of the donor elements of the molecule, such as carbazole.^60^ Similar oxidation potentials (as well as *E*_ox_^(onset)^ ≈ 0.63 V vs Fc/Fc^+^) were recorded for the tested compounds at about *E*_ox_ = 0.75 V vs Fc/Fc^+^ and the second irreversible oxidation peak also shows a similar value (*E*_ox_ about 1.40 V vs Fc/Fc^+^), indicating oxidation of the carbazole group (cf. Table 3(61)). The carbazole is known as a structure that can undergo polymerization.^19^ However, the carbazole is substituted at the 3N position, which may inhibit the polymerization process. This possibility was also checked, although no polymer formation was observed. The electrochemical investigations were also repeated using a GC (glassy carbon) working electrode in the DPV method. The differences between the potentials measured with the Pt electrode and the GC electrode were within the 0.1 V range. Based on the onset potentials, as mentioned above, the IP and EA energy levels were calculated. The IP values were similar for the investigated compounds (in the range of −5.75 to −5.67 eV), as well as the EA values in the case of tBuCBzSO_2_Ph and tBuCBzTCF (−4.10 to −3.97 eV). However, the EA values of tBuCBzSPh were higher (−3.79 to −3.72 eV). The presented compounds are characterized by a low energy band gap (2.02–1.65 eV), which is desirable in optoelectronic applications.

### Potential Applications

#### OLED Devices

**Figure 5:**
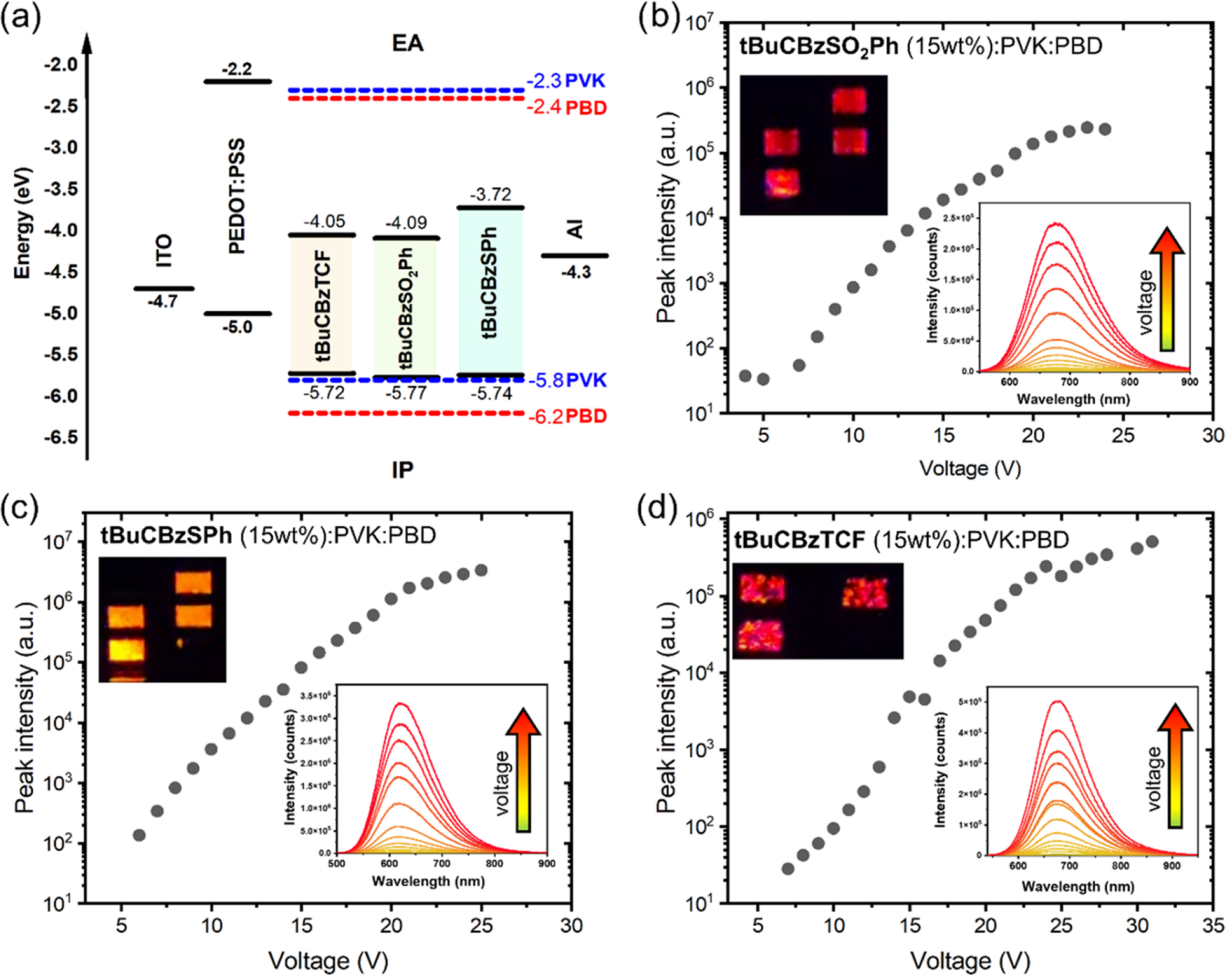
Energy diagram for all of the materials used in OLED devices and their typical design (a). Electroluminescence peak intensities of OLEDs vs applied voltage for tBuCBzSO_2_Ph (b), tBuCBzSPh (c), and tBuCBzTCF (d) doped on the PVK: PDB matrix at 15 wt %. Insets are showing the evolution of electroluminescence spectra upon increasing voltage and real photographs of OLED emission.

The investigated compounds were used as the active layer in the device structures ITO/PEDOT:PSS/compound/Al and ITO/PEDOT:PSS/compound:PVK:PBD/Al, the fabrication details of which can be found in the Experimental Section. The guest–host active layer structure was constructed with the three components: compound:PVK:PBD (PVK:PBD binary matrix ratio 50:50 wt %). The poly(9-vinylcarbazole) (PVK) and 2-(4-*tert*-butylphenyl)-5-(4-biphenylyl)-1,3,4-oxadiazole (PBD) were used respectively as a hole and an electron conductive material. The measurements were conducted for compound content in the active layer equal to 1, 2, and 15 wt %, as well as for neat-dye films sandwiched between the PEDOT:PSS layer coated on the ITO and Al layer. A schematic representation of the design of an OLED with ionization potentials and electron affinities of used materials is shown in Figure 5a. The presented diodes emit light from yellow (λ_EL_ = 600 nm), orange (λ_EL_ = 608–629 nm), and red (λ_EL_ = 638–716 nm) spectral regions under minimum external voltage between 6 and 10 V, depending on the OLED composition. The reason that underlies the utilization of high driving voltage for the studied compounds can be caused by the thickness of the layers and lack of the exciplex formation, which ensures a general carrier injection barrier or limitation resulting from molecular design.^62−64^ Based on the emission intensity vs applied voltage plots, we were able to identify the applicability of investigated compounds in the fabrication of OLEDs. Comparing Figures S6c and 5, we see a similar OLED performance for tBuCBzSPh doped to PVK:PBD in the amount of 15 wt % and a neat film. The highest electroluminescence intensity was observed for a device with a neat tBuCBzSPh and under an external voltage of 20 V (cf. Table 4); however, the device degrades at lower voltages. On the other hand, for moderate voltages (9–16 V), the emission intensity is higher for 15 wt % of dye in the PVK:PBD layer. In almost all cases, the tBuCBzSPh compound has shown superiority over other dyes in the performance of the OLED, as presented in Figures 5c and S6–S8.

**Table 4:** Electroluminescence Data^b^

	device parameters		
the active layer structure	λ_EL_^a^(nm)	EL_Max_^a^(counts)	*U*_EL,Max_^c^(V)
tBuCBzSO_2_Ph	716	354,827	26
tBuCBzSO_2_Ph(1 wt %):PVK:PBD	629	233,057	25
tBuCBzSO_2_Ph(2 wt %):PVK:PBD	608	9836	12
tBuCBzSO_2_Ph(15 wt %):PVK:PBD	675	242,598	23
tBuCBzSPh	655	4,805,200	20
tBuCBzSPh(1 wt %):PVK:PBD	600	597,191	26
tBuCBzSPh(2 wt %):PVK:PBD	600	8519	22
tBuCBzSPh(15 wt %):PVK:PBD	619	3,341,700	25
tBuCBzTCF	711	2192	15
tBuCBzTCF(1 wt %):PVK:PBD	638	233,743	31
tBuCBzTCF(2 wt %):PVK:PBD	654	5266	22
tBuCBzTCF(15 wt %):PVK:PBD	676	504,400	33

a λ_EL_ – maximum of the electroluminescence band.

b EL_Max_ – maximum intensity at λ_EL_.

c *U*_EL,Max_ – external voltage for the maximum electroluminescence intensity. The PVK:PBD ratio 50:50 wt %.

The exception from this observation can be noticed for the 2 wt % of dye loadings to PVK:PDB matrices, where the highest intensity was observed for tBuCBzSO_2_Ph dye. However, in this case, the device based on the tBuCBzSO_2_Ph compound degraded rapidly after crossing only the 12 V of applied voltage (cf. Figure S9 and Table 4). For 1 wt % of dye concentration, the devices with tBuCBzSO_2_Ph and tBuCBzSPh performed similarly, but finally, the second one was able to reach higher output intensity at higher voltage values, which is depicted in Figure S9 and in Table 4. In the case of tBuCBzSO_2_Ph and tBuCBzSPh compounds, a high loading at 15 wt % results in nearly identical performance of the devices fabricated using only a neat film of dyes. However, for tBuCBzTCF, the PVK:PBD matrix improves the performance of the OLED (cf. Table 4 and Figure S6d). Typically, what can be seen from Table 4, Figures 5, S7 and S9 is the red shift of emission spectra with increasing loadings, with the greatest one reported for neat films. A combination of this behavior and OLED performances indicates the positive influence of the AIE effect on electroluminescence.

The maximum wavelength of pristine PVK:PBD absorption (shown in Figure S4) is located at 410 nm. The large shifts in absorption and emission wavelengths for dye-doped matrices presented in Figures S4 and S5 and described in Table S2 suggest the energy transfer from the matrix to the host. The location of the estimated IP energy level of the compounds oscillates around the IP level of the matrix (cf. Figure 5a). Due to the location of the IP and EA of the compounds used in the presented devices, the energy transfer and the trapping mechanism may coexist.

Furthermore, we also observed that the performance of devices can be changed by the amount of dye dopant and the wavelength of emission. For example, in the case of tBuCBzSO_2_Ph and tBuCBzTCF, we are able to shift the emission into the biological window by increasing the dye concentration. For neat films, the emission from tBuCBzSPh is also in the biological window region. Notably, the emission can even reach the NIR range for other compounds.

#### Lasing

**Figure 6:**
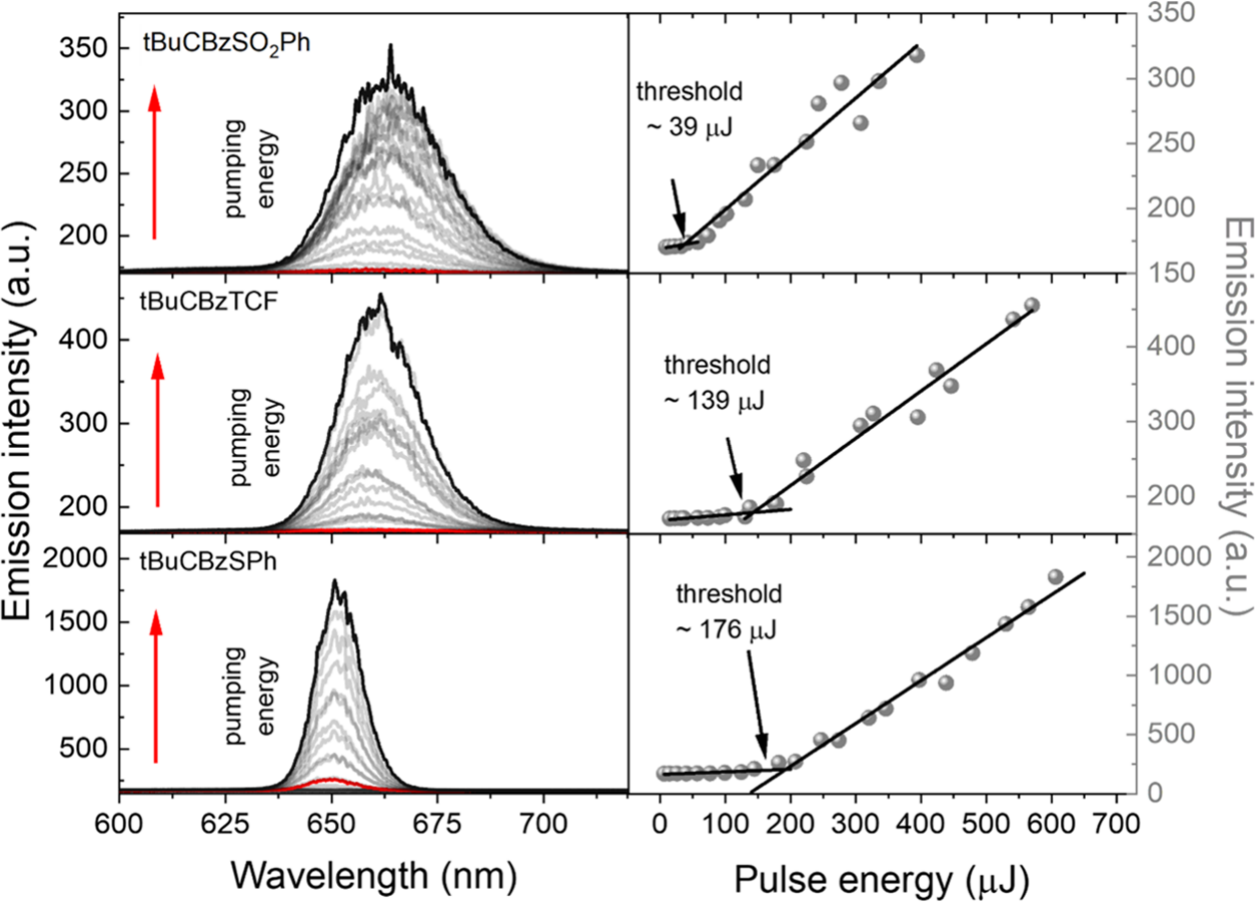
Evolution of the emission spectra with increasing pumping energy for PMMA layers doped with ∼2% of the studied compounds. Narrow modes typical for coherent RLng are visible for all samples (left panel), presented as a gray and transparent continuous line and black line at the top of the spectra related to the maximum of the utilized laser pump energy. Red lines represent the value of the laser threshold. Charts on the right show the corresponding peak intensity dependence on pumping energy with typical RLng inflection points indicating the lasing threshold.

The laser emission was investigated using a nanosecond laser-based optical system as described in the Experimental Section. For those measurements, we utilized polymeric asymmetric slab waveguides/quasiwaveguides made of poly(methyl methacrylate) (PMMA) deposited on microscopic support glass slides. Samples contained a constant concentration of each compound (∼2% dye with the polymer). All samples were prepared via the drop-casting method (see the Sample Preparation section). PMMA was chosen due to its well-known properties and good optical quality.^30^ The pumping wavelength was set to the maximum of the absorption spectra measured for the thin films for all examined compounds (λ^abs^tBuCBzSO_2_Ph = 503 nm, λ^abs^tBuCBzSPh = 455 nm, λ^abs^tBuCBzTCF = 490 nm—note that all of them are slightly red-shifted with respect to liquid solutions see Figure S10). As displayed in Figure 6, the emission spectra of all dyes are characterized by narrow and high spikes appearing in a random fashion after the pumping energy reaches the threshold of laser action. The visualization of this parameter was presented as a red line inside each emission spectra. The threshold value was calculated using a well-known method, namely the light–in–light–out (Li-Lo).^65^ The rapid growth of the emission intensity corresponds to the development of laser emission from the photoluminescence spectra, resulting in a significant increase in the emission intensity. These changes can be represented as points, and the two straight lines drawn through them, which intersect at the inflection point, represent the value of the random laser threshold. The described phenomenon is correlated with coherent random lasing.^66^ The formation of the spikes is associated with the interference effect of the light trapped inside the closed loop in the scattering media occurring in the sample. This effect enhances the amplification of specific modes, resulting in a narrow spike observation. The RLng was observed for all studied compounds at bands located at around ∼665 nm for tBuCBzSO_2_Ph and tBuCBzTCF and 655 nm for tBuCBzSPh. The full width at half-maximum (fwhm) of whole photoluminescence bands collapsed from approximately 126, 136, and 114 nm when threshold conditions were reached around 28.5, 22.0, and 12.6 nm, respectively. Typically, in organic dyes, a huge number of oscillatory states ensure the 4-level system of laser operation. The internal conversion speed within the ground electronic state determines between which states the population inversion can appear and thus determines the shape of the gain profile. Indeed, the polymeric layer with tBuCBzSPh shows the smallest fwhm of RLng; however, at this stage of research, it is impossible to determine whether this observation is due to the slowest internal conversion within the ground electronic state or to the limited waveguiding. The threshold conditions of laser emission in the case of the tBuCBzSO_2_Ph dopant were found to be around 39 μJ/pulse, which is the lowest reported in this study. The tBuCBzTCF doped system is characterized by an energy threshold of around 139 μJ/pulse, while for tBuCBzSPh, this value is equal to 176 μJ/pulse. Assuming the Gaussian shape of pulses, the mentioned values correspond to the following peak powers: 7.3, 26.1, and 33.1 kW.^67^

## Discussion

Theoretical calculations largely contribute to rationalizing the CT nature of the studied molecules: the changes in the calculated dipole moment vary between 16.70 and 23.59 D, depending on the molecule and its geometry. Interestingly, tBuCBzSPh exhibits significantly lower CT parameters, i.e., μ_ES_*-μ*_GS_ decreased to ∼17D with respect to the two other derivatives (cf. Table 2), which can be attributed to the large electron density depletion “orb” localized on the sulfur atom of the EDD plot. Therefore, the SPh moiety can be viewed as a weak secondary donor unit attached directly to the electron-accepting dihydrofuran unit, thus impeding the charge transfer along the molecular axis. As a result, it shows the smallest red shift of emission and molar extinction coefficient (cf. Table 1). For −SO_2_Ph and −TCF moieties, the CT character is rather similar, which is reflected in nearly identical calculated μ_*ES*_*-μ*_*GS*_ values as well as comparable positions of the absorption and emission spectra.

The very strong CT character of the investigated molecules leads to a significant red-shifted emission. Particularly, for the −SO_2_Ph and −TCF compounds, emission reaches the short-wavelength edge of the biological window. Moreover, the formation of the aggregates can cause a further red-shift of the photoluminescence, as was reported for pure dye films (reaching the NIR region) and PVK:PBD matrices with the highest dye loadings (15%). Consequently, those two compounds are even better suited for biological applications.

Furthermore, all of the investigated dyes exhibit AIE behavior. In general, the issues associated with quenching during aggregation are common problems with the implementation of organic dyes, especially in the biological field. For instance, protein aggregation is correlated with numerous human diseases, such as cancers and neurodegenerative or metabolic disorders.^68^ Using AIE-based organic dyes, such as those presented in this study, to monitor unwanted protein changes using various methods can overcome the mentioned limitations and lead to increased efficiency in detecting pathological changes within the organism. Nevertheless, the existence of aggregates can shift the emission toward biological windows and significantly increase the emission intensity, facilitating potential future applications in medical sciences.

As was mentioned before, all the compounds show relatively small band gap values, which is a frequently desired feature for the construction of OLEDs. Several types of OLED devices were prepared. In general, a higher value of the electroluminescence peak was obtained for the tBuCBzSPh compound. It is worth mentioning that recently, the intensity of the emission was proposed as a significant property for the use of OLEDs, especially when we consider the transmission of light through the skin. As was previously reported, for red OLEDs, the transmitted light was approximately 46% of the initial intensity of electroluminescence.^69^ The mentioned parameter can be beneficial in various types of advanced and smart wearables containing OLEDs, such as in cutaneous wound healing^61^ or smart textiles as a proposition for clothing capable of responding to emergencies or providing ubiquitous healthcare.^70^ On the other hand, the weakest performance was reported for the TCF moiety, which is in accordance with emission quantum yield measurements described by Rémond et al. for solid-state emission.^48^ This observation once again shows that the AIE effect can play a beneficial role in optoelectronic device construction, which is required for biological applications. The investigated dyes can perfectly match the wavelengths desired for the development of novel, organic-based pulse oximeters^10^ or as a light source for stimulating gene expression for tumor suppression, which has a positive impact on the aging pathway.^71^

Finally, we observed RLng emission from dye-doped polymeric layers at wavelengths that can be linked to the emission of aggregates. The random nature of laser emission is possibly the result of the enchanted light scattering from aggregates as well as layers’ irregularities (characteristic of the drop-casting technique). The energy thresholds were estimated to be moderate for organic dyes; however, there is still room for further optimization, for example, by applying different types of high-quality resonators or by optimizing the polymeric system (thickness, type of polymer, dye concentration, etc.). However, the threshold conditions shown here are still relatively low for obtaining lasing, for example, by using a cheap DPSS laser operating in the pulse regime as pumping sources. It is worth noting that the laser threshold value is a crucial parameter of any laser device’s performance. For instance, besides the abovementioned application of the DPSS laser, a low laser threshold stands for the ability to operate with less input pump power; thus, it can be beneficial, for example, in the application where the value of the used power pump is crucial in advanced optoelectronic devices,^72^ portable sensors,^73^ microlasers,^74^ or even as a part of the integrated OLED devices as a pumping electrically driven organic semiconductor laser.^75^

In general, the energy threshold level is an intricate function related to rate equations, dependent on emission quantum yield, emission lifetime, absorption coefficients, speed of different transitions, resonator quality factor, etc. Therefore, the observed threshold conditions cannot be simply explained in terms of the emission quantum yields. However, we can see some coincidences with the extinction coefficient. For example, the lowest threshold was reported for tBuCBzSO_2_Ph, for which we observe the highest ε and moderate emission quantum yield. Next, a slightly higher threshold was reported for tBuCBzTCF. In this case, ε is slightly lower than that for tBuCBzSO_2_Ph, but its quantum yield of emission is the lowest.^17^ Finally, for tBuCBzSPh, the threshold conditions are the highest. This behavior can be explained in terms of the relationship between the absorption and gain coefficients. First, the dye with the lowest ε cannot absorb as much light as other dyes; thus, the pumping is less efficient. Second, as the gain coefficient can be seen as a negative absorption coefficient, the potential gain is determined by absorption. Therefore, the interplay between the ε value and emission quantum yields can qualitatively explain the observed coincidence.

Finally, it is worth highlighting that the emission range located between 600 and 700 nm is reported in the literature as an ideal wavelength, for instance, for photodynamic therapy (PDT) due to its deep tissue penetration while simultaneously avoiding overlap with the absorption peaks of water and oxygenated hemoglobin.^76^ In this context, OLEDs having electroluminescence in the red region of the light are suitable for all purposes correlated with the PDT, where light and a specialized photosensitizer are used to create a reactive form of oxygen capable of killing bacteria in its surroundings.^77,78^ On the other hand, RLs are also well-known for their application in medicine, as was previously mentioned. For example, RLs can detect early-stage tumors or can be used in the field of automated laser surgery systems.^79^

## Conclusions

In conclusion, the investigated dyes show a remarkable red shift in emission due to the ICT effect and formation of aggregates. The OLED emission and RLng can be obtained in the NIR region of light, indicating the usefulness of the investigated dyes for biological purposes. The presence of the AIE phenomenon allowed more flexible functionalization of guest–host systems using higher loads of dye-dopants as well as designing devices based only on neat-dye films, highly influencing the optical properties of the resulting system. Thus, by simple concentration changes, it is possible to tune the emission properties of the resulting OLED device. In both cases (OLEDs and RL), we obtained significant luminescence intensities for wavelengths characteristic of the biological window, which paves the way for the further utilization of dyes described here in biomedical research and applications. Finally, there is still room for optimizing RL samples and the design of an OLED to improve the performances of both types of devices.

## Supporting Information Available

Additional data, and a table with more comprehensive information, facilitating a deeper understanding of the study’s findings (PDF)

## Abbreviations

OLED: organic light-emitting diodes
RL: random laser
RLng: random lasing
OFET: organic field effect transistors
PSC: organic–inorganic hybrid perovskite solar cell
ACQ: aggregation-caused quenching
AIE: aggregation-induced emission
ICT: intramolecular charge transfer
D-π–*A*: donor-π–*a*cceptor
NIR: near-infrared
TD-DFT: time-dependent density functional theory
SI: Supporting Information
THF: tetrahydrofuran
EED: electron density difference
Δ*G*: Gibbs free energy
CT: charge transfer
AE: absolute errors
μ_*ES*_*-μ*_*GS*_: change in the dipole moment
*d*_CT_: charge transfer distance
*q*_CT_: total transferred charge
f_w_/%: water fraction
∫I^emi^dλ: integrated emission spectra
DSC: differential scanning calorimetry
*T*_m_: melting temperature
*T*_g_: glass transition temperature
*T*_cc_: crystallization temperature
CV: cyclic voltammetry
DPV: differential pulse voltammetry
IP: ionization potentials
EA: electron affinities
Pt: platinum wire
*E*_ox_: first oxidation process
E_red_: first reduction process
E_red_^(onset)^: onset potential of the first reduction process
*E*_ox_^(onset)^: onset potential of the first oxidation process
GC: glassy carbon working electrode
PVK: poly(9-vinylcarbazole)
PBD: 2-(4-tert-butylphenyl)-5-(4-biphenylyl)-1,3,4-oxadiazole
PMMA: poly(methyl methacrylate)
fwhm: full width at half maximum
