# Supplementary material for: Emission in the Biological Window from AIE-Based Carbazole-Substituted Furan-Based Compounds for Organic Light-Emitting Diodes and Random Lasers
Source: ACS Omega. 2024 Sep 18;9(39):40769–82. doi: 10.1021/acsomega.4c05484 (PMC11447741; doi:10.1021/acsomega.4c05484)
Supplement: Supplementary file 1 — ao4c05484_si_001.pdf [file ao4c05484_si_001.pdf]

Electronic Supporting Information

Emission in the biological window from AIE-based carbazole-substituted furan-based compounds for organic light-emitting diodes and random lasers

Kamila Lupinska[a], Sonia Kotowicz[b], Anna Grabarz[a,c], Mariola Siwy[d], Karolina Sulowska[a,e], Sebastian Mackowski[e], Lulu Bu[f], Yann Bretonnière[f], Chantal Andraud[f], Ewa Schab-Balcerzak[b,c], Lech Sznitko[a*]*

[a] Institute of Advanced Materials, Faculty of Chemistry, Wroclaw University of Science and Technology, Wybrzeze Wyspianskiego 27, 50-370 Wroclaw, Poland.

[b] Institute of Chemistry, University of Silesia, 9 Szkolna Str., 40-006 Katowice, Poland

[c] Department of Physical and Theoretical Chemistry, Faculty of Natural Sciences, Comenius University, Ilkovičova 6, 84215 Bratislava, Slovakia

[d] Centre of Polymer and Carbon Materials, Polish Academy of Sciences, 34 M. Curie-Sklodowska Str., 41-819 Zabrze, Poland

[e] Institute of Physics, Faculty of Physics, Astronomy and Informatics, Nicolaus Copernicus University, 5 Grudziadzka Str., 87-100 Torun, Poland

[f] Univ Lyon, Ens de Lyon, CNRS UMR 5182, Laboratoire de Chimie, F69342, Lyon, France

* kamila.lupinska@pwr.edu.pl; lech.sznitko@pwr.edu.pl

Contents: **page**

1. Quantum chemical calculations – results 3
2. Aggregation impact on photoluminescence 3
3. Thermal investigation - results 4
4. Electrochemical investigations – results 5
5. Optical properties of layers 6
6. OLED performances 9
7. Absorption of thin polymer films 13

1. Quantum chemical calculations – results

Table S1 *Gibbs energy between two stable conformers and the probability of the most stable conformer presence at room temperature according to Boltzmann distribution.*

	Compound	ΔG (kcal/mol)	Norm. fraction*
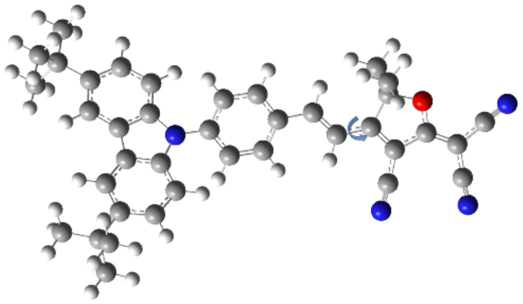	tBuCBzSO$_2$Ph	2.72	0.99
	tBuCbzSPh	0.47	0.69
	tBuCbzTCF	0.08	0.53

*Population distribution of conformers at room temperature, given fraction corresponds to most stable conformers.

2. Aggregation impact on photoluminescence

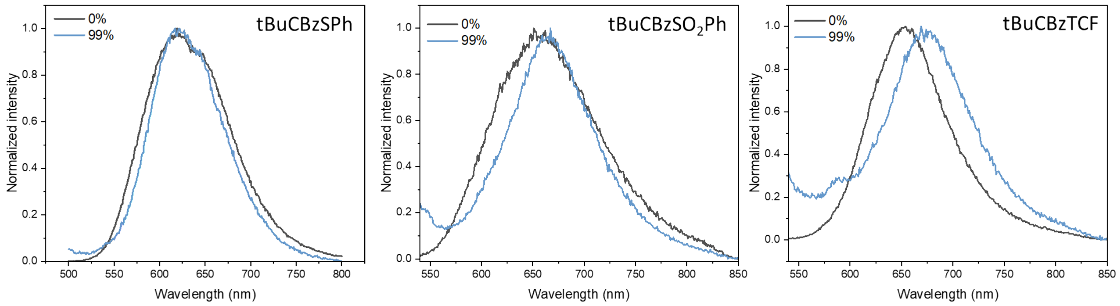

Fig. S1 Impact of water on emission spectra shapes and positions.

3. Thermal investigation - results

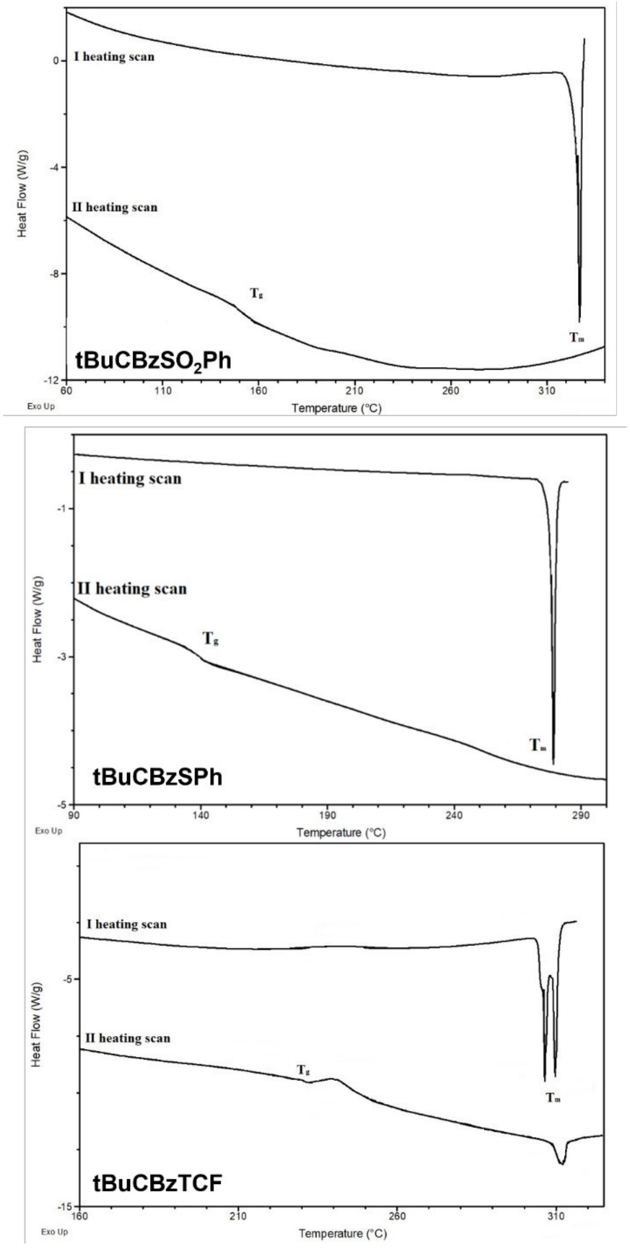

Fig. S2 DSC curves.

4. Electrochemical investigations – results

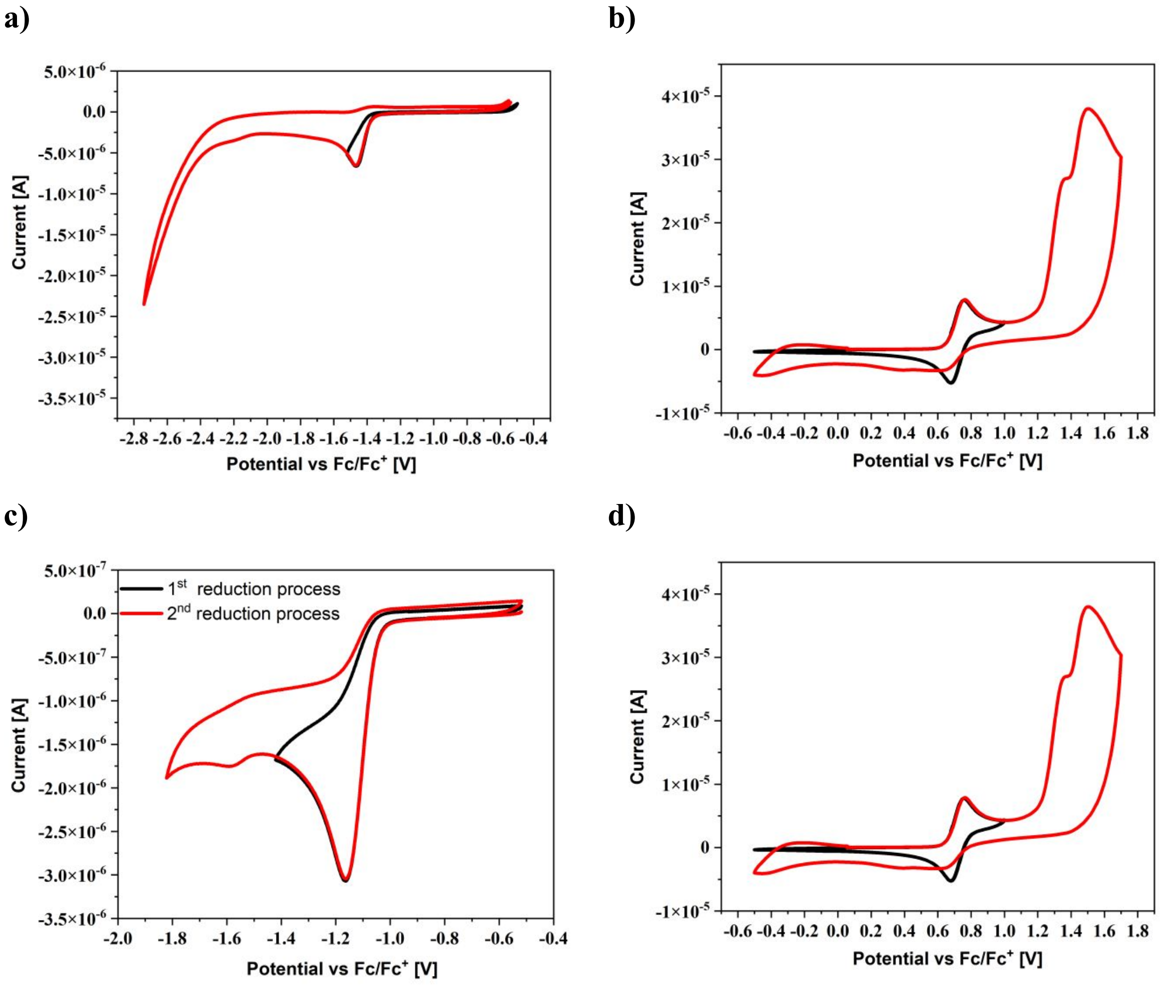

Fig. S3 The cyclic voltammograms of the (a,b) tBuCBzSPh and (c,d) tBuCBzTCF.

((a,c) – the reduction processes, (b,d) – the oxidation processes).

5. Optical properties of layers

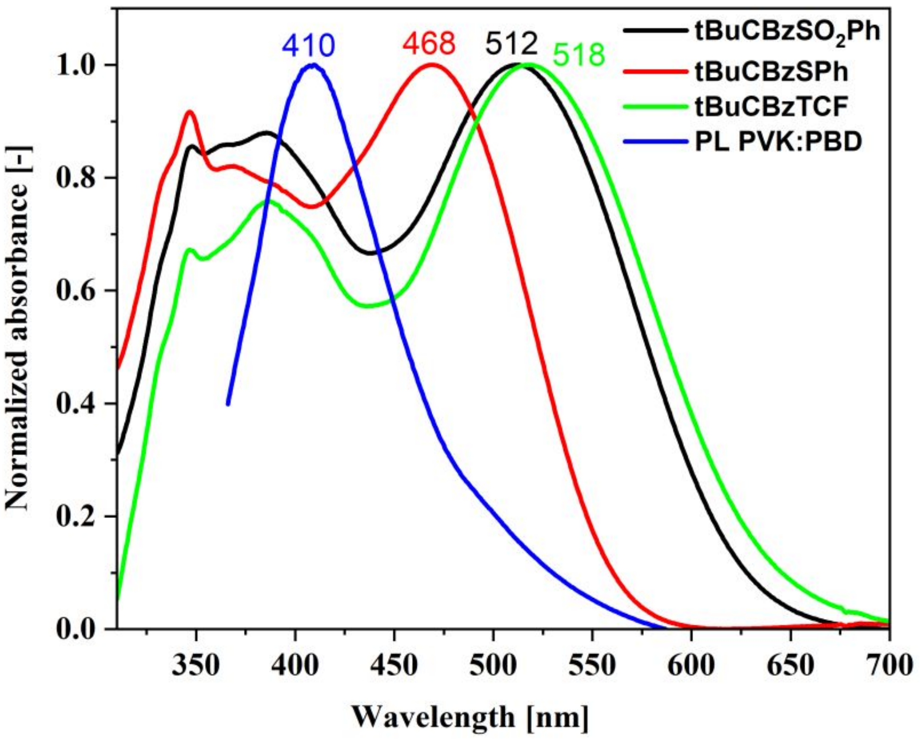

Fig. S4 The absorption spectra of samples in the form of thin films obtained from a homogeneous chloroform solution (10 mg/ml) on a glass substrate.

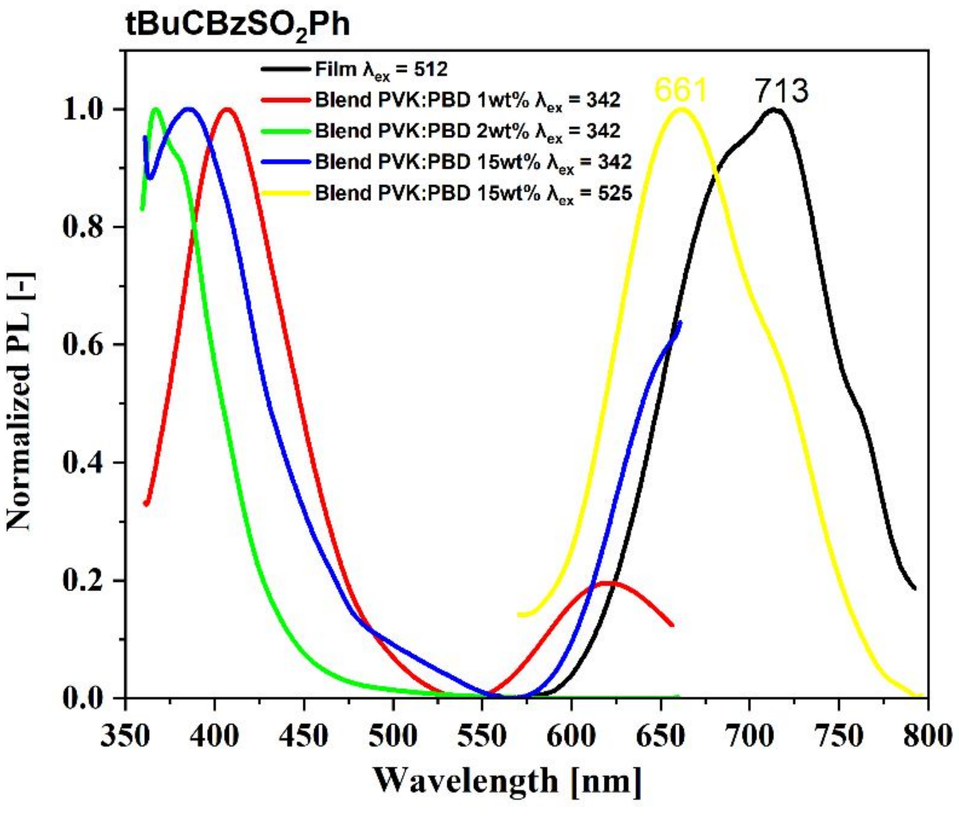

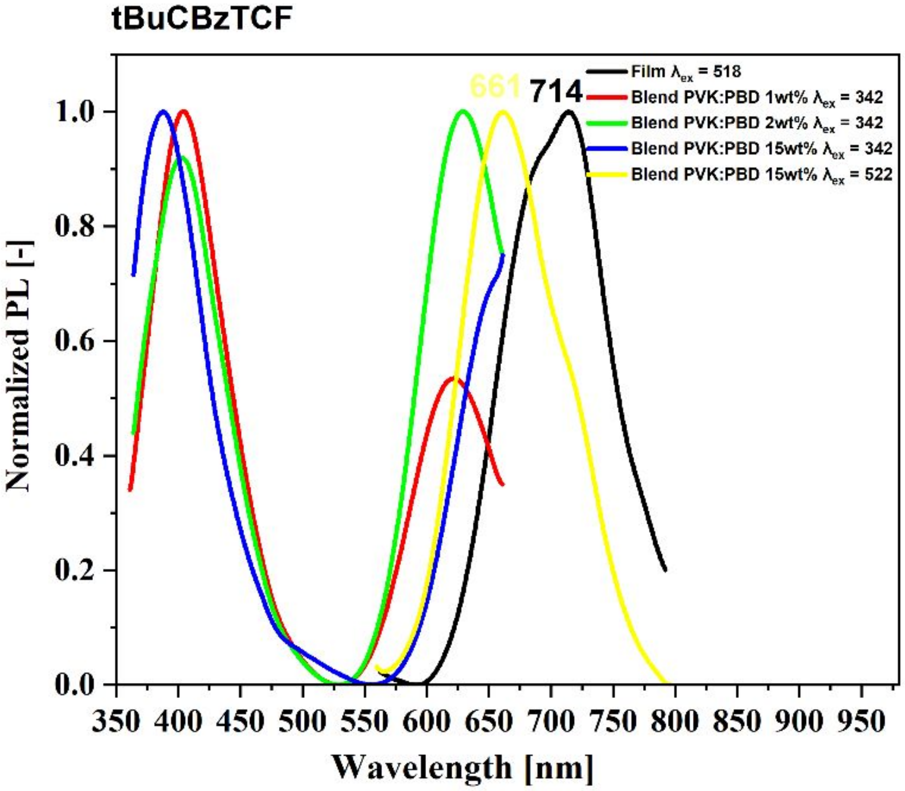

Fig. S5 The normalized PL spectra of the tBuCBzSO$_2$Ph and tBuCBzTCF compounds in the form of thin films and blends with binary PVK:PBD matrix.

Table S2 *The UV-Vis and PL data in various media of the investigated compounds.*

**Compounds**	**Medium**	**UV-Vis** λ_{max} **[nm]**	**PL** λ_{em} **[nm]**
**tBuCBzSO₂Ph**	THF	489	653
	FILM	347,385,512	713
	PVK:PBD 1wt%	310^{sh},342^{sh},529	no PL
	PVK:PBD 2wt%	310^{sh},342^{sh}	-
	PVK:PBD 15wt%	310^{sh},342^{sh}, 385,525	661
**tBuCBzSPh**	TFH	453	618
	FILM	346,369,468	631
	PVK:PBD 1wt%	310^{sh},342^{sh}	-
	PVK:PBD 2wt%	310^{sh},342^{sh}	-
	PVK:PBD 15wt%	310^{sh},342^{sh},479	601
**tBuCBzTCF**	THF	487	649
	FILM	346,387,518	714
	PVK:PBD 1wt%	310^{sh},342^{sh}	-
	PVK:PBD 2wt%	310^{sh},342^{sh}	-
	PVK:PBD 15wt%	310^{sh},342^{sh}, 387,522	661
**PVK:PBD**	FILM	310^{sh},342^{sh}	410

. ^{sh} – shoulder. No PL – lack of emission. The λ_{ex} band has been underlined.

6. OLED performances

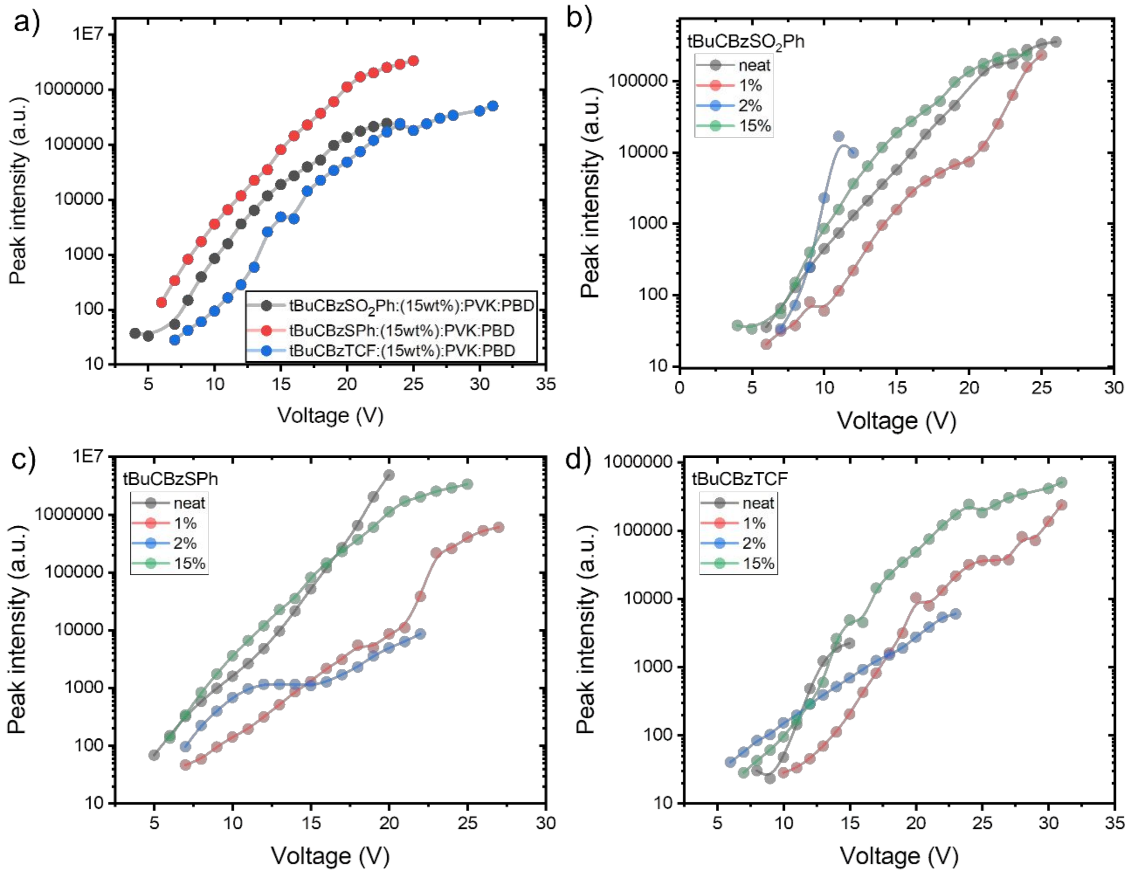

Fig. S6. Comparison of electroluminescence peak intensity vs. applied voltage for OLED with 15wt% of dye dopant to PVK: PBD matrix a). Influence of dye concertation on OLEDs performance for tBuCBzSO$_2$Ph b), tBuCBzSPh c) and tBuCBzTCF d) compound.

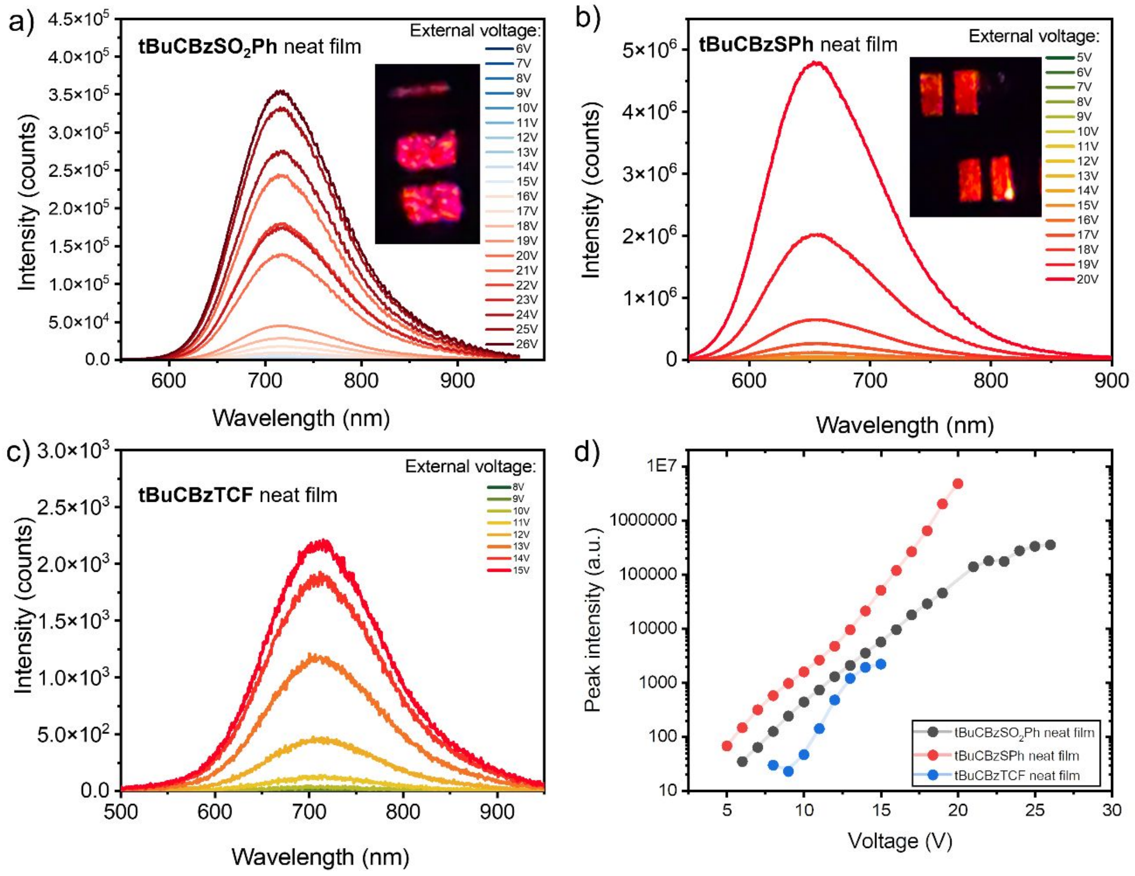

Fig. S7. The electroluminescence spectra recorded for the tBuCBzSO$_2$Ph a), tBuCBzSPh b) and tBuCBzTCF c) neat films and corresponding to them luminescence emission intensity vs. applied voltage dependencies d). Insets show a photograph of OLEd's electroluminescence.

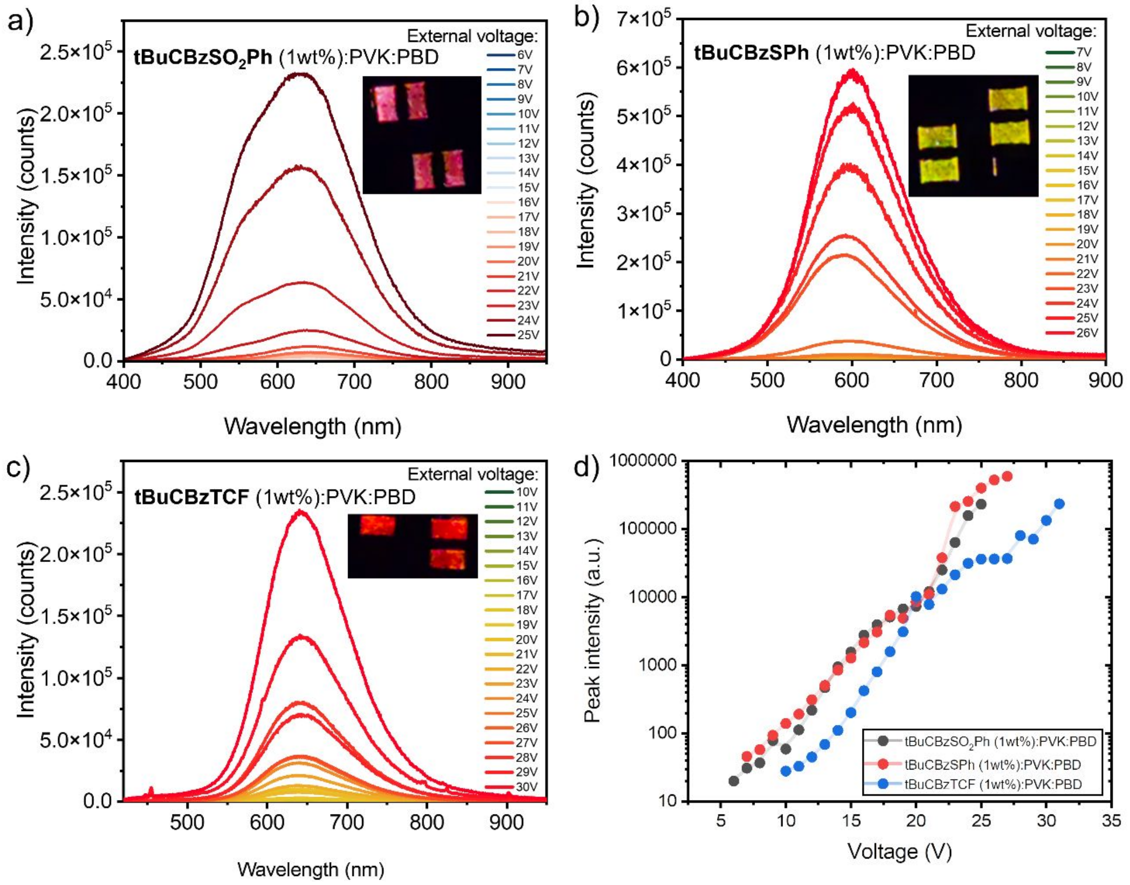

Fig. S8. The electroluminescence spectra recorded for the tBuCBzSO$_2$Ph a), tBuCBzSPh b) and tBuCBzTCF c) dyes, doped to PVK:PDB matrix in 1wt%, and corresponding to them luminescence emission intensity – applied voltage dependencies d). Insets show a photograph of OLEd's electroluminescence.

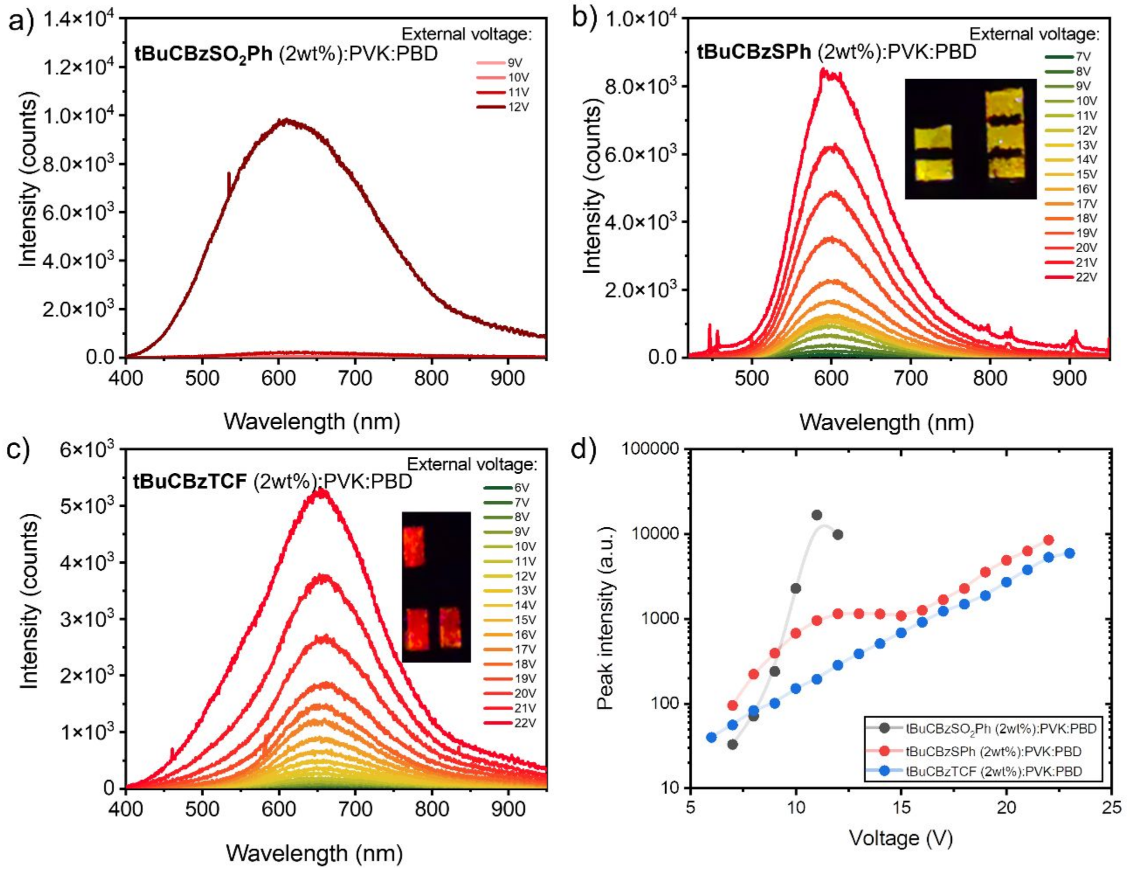

Fig. S9. The electroluminescence spectra recorded for the tBuCBzSO$_2$Ph a), tBuCBzSPh b) and tBuCBzTCF c) dyes, doped to PVK:PDB matrix in 2wt%, and corresponding to them luminescence emission intensity – applied voltage dependencies d). Insets show a photograph of OLEd's electroluminescence.

7. Absorption of thin polymer films

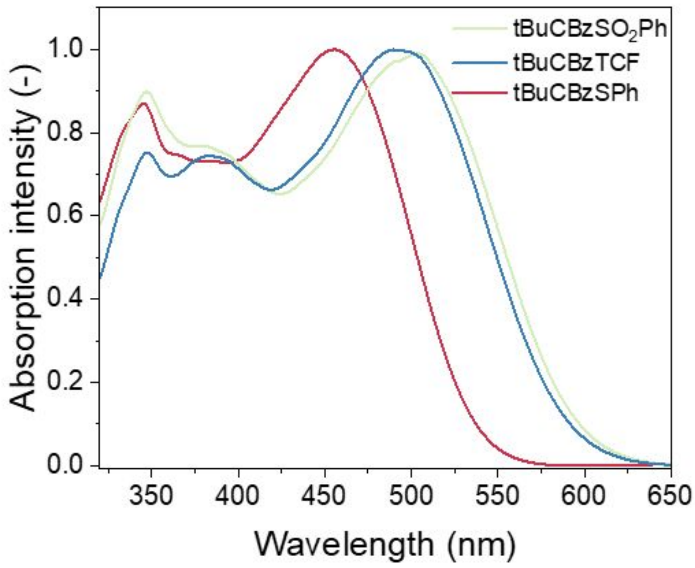

Fig. S10. Absorption spectra measured for thin polymer films doped with 2% of selected dye in order to PMMA.
